# Whole-Genome Sequences of Two *Staphylococcus aureus* ST398 Strains of Human Origin, S94 and S100

**DOI:** 10.1128/genomeA.00691-13

**Published:** 2013-08-29

**Authors:** Anna-Rita Corvaglia, Patrice François, Xavier Bertrand, Roland Quentin, David Hernandez, Nathalie van der Mee-Marquet

**Affiliations:** Genomic Research Laboratory, University of Geneva Hospitals, Geneva, Switzerlanda; UMR 6249 Chrono-environnement, Université de Franche-Comté, Besançon, Franceb; Service de Bactériologie et Hygiène, Centre Hospitalier Régional Universitaire, Tours, Francec; Réseau des Hygiénistes du Centre, Centre Hospitalier Universitaire, Tours, Franced

## Abstract

Sequence type 398 (ST398) *Staphylococcus aureus* was originally associated with animal infection. We announce the complete genome sequences of two ST398 methicillin-susceptible *S. aureus* strains of human origin, S94 and S100. The genome sequences assist in the characterization of interesting ST398 features related to host specificities*.*

## GENOME ANNOUNCEMENT

*Staphylococcus aureus* sequence type 398 (ST398) was originally described in livestock (1) but is now recognized as a causative agent of severe infections in human. We have recently reported the emergence of specific clades of ST398 showing specific bacteriophage content and appearing adapted to humans (2). We report here the genome sequences of two *S. aureus* strains responsible for severe infection in humans in an animal-free context.

*S. aureus* S94 and S100 were recovered in 2009 and 2010, respectively, from bloodstream infections in two patients hospitalized in two distant regions of France. Both patients had no contact with livestock animals. Purified genomic DNA was subjected to whole-genome shotgun sequencing by using MiSeq and HiSeq 2000 systems (Illumina, Inc.) for S94 and S100, respectively. Following fragmentation, end reparation, and sample tagging, the sequencers produced 10.1 and 11.5 million 100-bp paired-end reads, yielding appreciable coverage of around 375× and 426× for strains S94 and S100, respectively. Assembly was performed using Edena 3.0 (3) and resulted in 54 and 43 contigs for strains S94 and S100, respectively. The larger contigs showed sizes of 998,000 base pairs (bp) for strain S100 and 338,000 bp for strain S94. Overall assembly values were satisfactory (total for strain S94, 2.69 Mbp, *N*_50_, 202,000 bp; strain S100, 2.74 Mbp, *N*_50_, 338,000 bp). In strains S94 and S100, a total of 2,467 and 2,551 predicted coding sequences (CDS), respectively, were detected by rapid annotations using RAST subsystems technology (4). Using CD-HIT (5), we found that the majority of genes (*n* = 2,427) were common to both strains (identity >80%). More than 54% of the genes were assigned to specific subsystem categories by RAST (4). In addition to CDS, RAST identified 82 structural genes (59 tRNA and 23 rRNA genes) in strain S94 and 79 structural genes (59 tRNA and 20 rRNA genes) in strain S100. Note that strain S100 is devoid of plasmid, whereas S94 harbors a 5.54-kb plasmid.

The two *S. aureus* genomes contain known virulence factors, such as pore-forming hemolysins and bacterial adhesins as well as similar prophages, including the immune evasion cluster containing the *scn* and *chp* genes, known to interact with host immunity. Annotation confirmed the absence of resistance determinant genes. Interestingly, both strains are missing a type IV restriction system but harbor a type I restriction modification system showing a sequence similar to that of the functional one sequenced in the reference isolates, as well as a putative type III system of bacteriophage origin.

We conclude that these *S. aureus* ST398 genome sequences contain specific features supporting the observed tropism and genome plasticity of these bacteria.

### Nucleotide sequence accession numbers.

The whole-genome sequences of *S. aureus* S94 and S100 were deposited in the DDBJ/EMBL/GenBank databases under the accession numbers AUPW00000000 and AUPV00000000, respectively.
